# α-Farnesene production from lipid by engineered *Yarrowia lipolytica*

**DOI:** 10.1186/s40643-021-00431-0

**Published:** 2021-08-23

**Authors:** Yinghang Liu, Zhaoxuan Wang, Zhiyong Cui, Qingsheng Qi, Jin Hou

**Affiliations:** 1 State Key Laboratory of Microbial Technology, Shandong University, Binhai Road 72, Qingdao, 266237 People’s Republic of China; 2 CAS Key Lab of Biobased Materials, Qingdao Institute of Bioenergy and Bioprocess Technology, Chinese Academy of Sciences, Qingdao, 266101 People’s Republic of China

**Keywords:** α-Farnesene, Erg12, Oleic acid, Waste cooking oils, *Yarrowia lipolytica*

## Abstract

Producing high value-added products from waste lipid feedstock by microbial cell factory has great advantages to minimize the pollution as well as improve the economic value of wasted oils and fats. *Yarrowia lipolytica* is a non-conventional oleaginous yeast and can grow on a variety of hydrophobic substrates. In this study, we explored its ability to synthesize α-farnesene, an important sesquiterpene, using lipid feedstock. Based on the α-farnesene production strain, we constructed previously, we identified that Erg12 was the key limiting factor to further increase the α-farnesene production. The α-farnesene production was improved by 35.8% through increasing the copy number of *ERG12* and *FSERG20* on oleic acid substrate. Expression of heterologous VHb further improved α-farnesene production by 12.7%. Combining metabolic engineering with the optimization of fermentation conditions, the α-farnesene titer and yield reached 10.2 g/L and 0.1 g/g oleic acid, respectively, in fed-batch cultivation. The α-farnesene synthesis ability on waste cooking oil and other edible oils were also explored. Compared with using glucose as carbon source, using lipid substrates obtained higher α-farnesene yield and titer, but lower by-products accumulation, demonstrating the advantage of *Y. lipolytica* to synthesize high value-added products using lipid feedstock.

**Supplementary Information:**

The online version contains supplementary material available at 10.1186/s40643-021-00431-0.

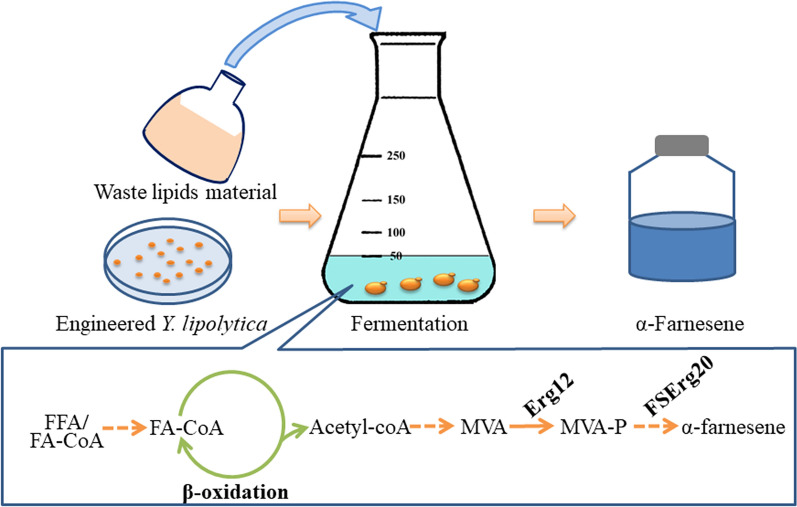

## Introduction

Farnesene is a simple acyclic sesquiterpene of the terpenoids family, and it is an important component of plant essential oils in nature (Miyazawa and Tamura 2007). At present, farnesene plays an important role in industry, agriculture and daily life. For example, in agriculture, farnesene, as a pheromone, can interfere in the normal feeding behavior and physiological process of aphids to avoid aphid invasion to crops (Hatano et al. 2010; Su et al. 2006). In industrial production, farnesene is used in lubricants, surfactants and cosmetics due to its superior properties (Armelle et al. 2014). In addition, farnesene has been identified as an important substitute of jet fuel due to its high carbon density and storage safety (Leavell et al. 2016). Farnesene also can be used as precursor of vitamin E, which promotes the industrial synthesis of vitamin E (Ma et al. 2020). With the increasing demand of farnesene, extracting farnesene from plants can no longer meet the demand, which encouraged researchers to develop more sustainable processes to produce farnesene. The most attractive one is through engineering microbial cell factories. Through metabolic engineering strategies, farnesene has been synthesized in different microorganisms. For example, the titer of β-farnesene reached 8.74 g/L using glycerol as carbon source in *E. coli* (You et al. 2017). Using *S. cerevisiae* as host, the highest titer of 130 g/L was achieved in the industrial production level (Meadows et al. 2016). Using the photosynthesis of cyanobacteria, CO_2_, H_2_O and light energy were converted to α-farnesene, and the production reached 4.6 ± 0.4 mg/L in 7 days (Lee et al. 2017). Previously, we constructed a mevalonate overproduction strain in *Yarrowia lipolytica*, and based on this strain, the strain producing 25.55 g/L α-farnesene was achieved by overexpressing the downstream α-farnesene synthesis pathway (Liu et al. 2019b). Therefore, microbial production of farnesene or other terpenoids shows attractive potential and prospects.

*Y. lipolytica* is a non-conventional oleaginous yeast and can grow on a variety of substrates including alkanes, fatty acids and oils (Nicaud 2012). It has strong adaptability to environmental stresses and robustly grows at a wide range of pH and salinity (Bankar et al. 2009). These characteristics make *Y. lipolytica* widely used in metabolic engineering to produce high value-added products (Miller and Alper 2019). Importantly, *Y. lipolytica* is able to directly grow on lipid feedstock such as plant oils or animal fats. It can synthesize 16 lipases, which are used to decompose intracellular and extracellular lipids for metabolic activities (Syal and Gupta 2017). It has strong β-oxidation, which provides abundant acetyl-CoA precursors for chemicals synthesis. Compared with glucose substrate, *Y. lipolytica* can accumulate more biomass (Worland et al. 2020) and has higher theoretical conversion rate of products in lipid substrates. Taking α-farnesene as an example, the theoretical conversion rate is 0.723 g/g oleic acid and 0.252 g/g glucose, respectively. Based on these advantages, many studies have used lipid substrates in the production of compounds in *Y. lipolytica*. For example, Li et al. (2020) used safflower oil for astaxanthin production and attained 167 mg/L in *Y. lipolytica*. In the meanwhile, 48% of astaxanthin was extracted to the extracellular by safflower oil, which is helpful to alleviate the toxicity of astaxanthin to cells. The best β-carotene titer (121  ±  13 mg/L) was achieved on canola oil-containing yeast–peptone medium, which was twofold higher than the glucose based yeast–peptone medium (Worland et al. 2020). On the other hand, due to its strong lipid utilization ability, more and more attention has been paid to the use of *Y. lipolytica* to transform waste lipid produced in industry, agriculture and daily life, such as waste cooking oils (WCO), olive oil processing wastes, and industrial derivative of animal fat, into high value-added products (Kamzolova et al. 2005; Liu et al. 2015, 2019a; Papanikolaou et al. 2002; Sarris et al. 2017). This has great benefits in environmental protection and economic performance improvement.

In this study, we optimized the previously constructed strain, producing α-farnesene from glucose (Liu et al. 2019b). For further improving the ability of recombinant strain to produce α-farnesene from low-cost oleic acid, we identified the key limiting steps of α-farnesene synthesis, increased the copy number of genes in metabolic pathway and optimized the oxygenation utilization capacity for better use of oleic acid. Combined with the optimization of fermentation conditions, the α-farnesene synthesis potential of the recombinant strain has been explored. The potential of the engineered strain to synthesize α-farnesene on WCO and other common edible oil substrates was also explored. This work will provide strategic insights for the development of *Y. lipolytica* as a microbial cell factory for the sustainable and economic production of valuable chemicals from waste oil materials.

## Results and discussion

### Comparison of α-farnesene synthesis capability on glucose and oil carbon sources

**Fig. 1:**
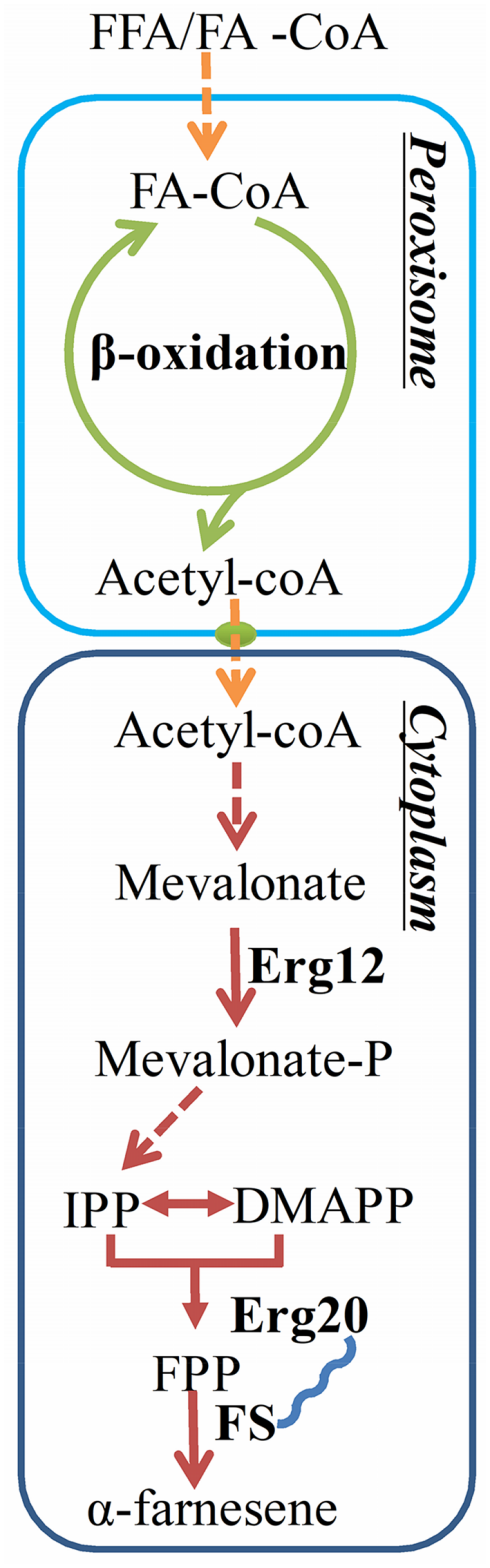
Metabolic pathway of α-farnesene synthesis from lipid substrate in *Y. lipolytica*. Lipid was activated to FA-CoA, which converts to acetyl-CoA through β-oxidation. Acetyl-CoA was transported to cytoplasm and α-farnesene synthesized via the MVA pathway. Here, Erg20 and FS were used by a linker with GGGS amino acid sequence. *FFA* free fatty acid; *FA-CoA* fatty acid-CoA; Black bold font, overexpression of proteins

In our previous study, we constructed the α-farnesene production of *Y. lipolytica* strain F5 by overexpression of all genes of the MVA pathway from acetyl-CoA to farnesyl pyrophosphate (FPP) and integration of *FSERG20* into genome twice by NHEJ-mediated genome integration, which accumulated 25.55 g/L α-farnesene with glucose as carbon source. Although a relatively high titer was achieved, the α-farnesene yield was still low. We also detected considerable accumulation of by-products such as citric acid, mannitol and mevalonate (MVA) in the final fermentation broth. *Y. lipolytica* has a vigorous metabolic activity of β-oxidation, and can decompose lipid into acetyl-CoA, the precursor of MVA pathway for α-farnesene production (Du et al. 2016; Fig. 1). Using lipid as carbon source may be more beneficial to avoid by-product synthesis and provide abundant precursor for synthesis of α-farnesene.

**Fig. 2:**
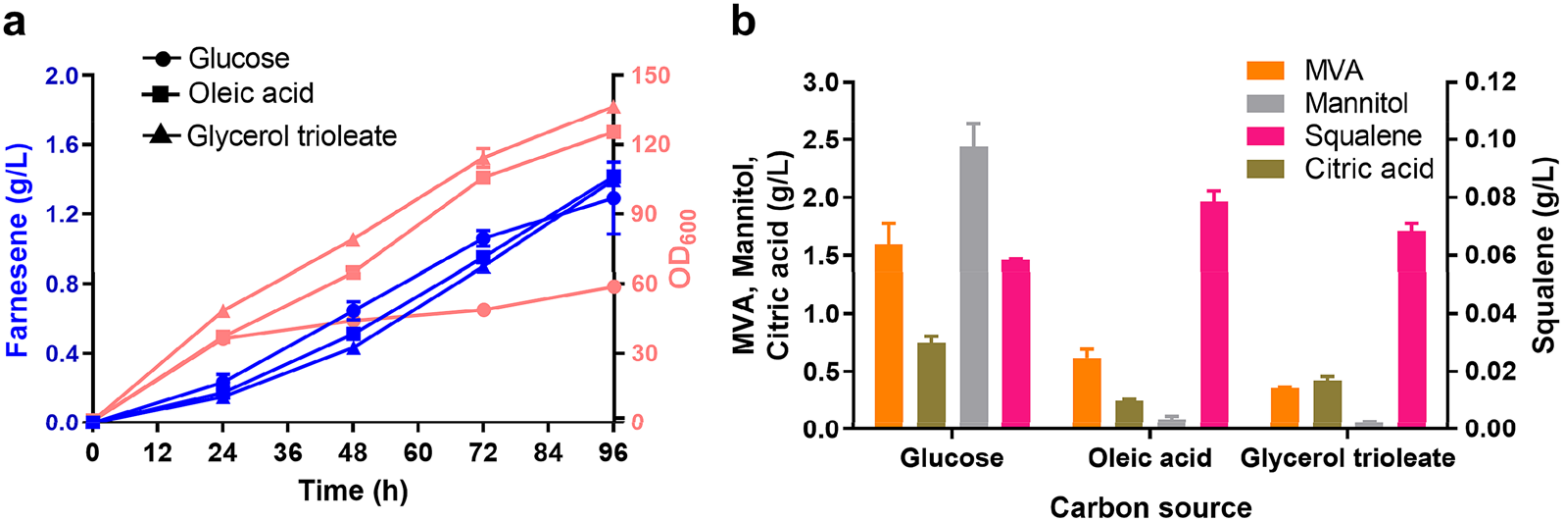
OD_600_, α-farnesene, and other metabolites production on different substrates by the F5 strain. **a** α-Farnesene and OD_600_ accumulation curve during fermentation. **b** Citric acid, mannitol, squalene and mevalonate accumulation. The metabolites were detected at 96 h fermentation in 300-mL shake flasks containing 50 mL YP medium with different carbon sources. Data represent the mean  ±  SD of biological triplicates

We first compared the growth, α-farnesene synthesis ability and other products’ accumulation of F5 strain on glucose carbon source and lipid carbon source, including oleic acid and triglyceride. We found that the accumulation of α-farnesene increased with the extension of fermentation time in YP medium with different carbon sources, and the titer reached 1.29 g/L, 1.42 g/L and 1.39 g/L on glucose, oleic acid and triglyceride, respectively, at 96 h (Fig. 2A). The titer of α-farnesene on lipid substrates was slightly higher than that on glucose substrate. In addition, it can be clearly observed that the OD_600_ reached 125 and 137 on oleic acid and triglyceride, respectively, which was 1.1-fold and 1.3-fold higher than that of 59 on glucose. This is consistent with previous reports that the lipid substrate can promote biomass accumulation (Papanikolaou et al. 2007). The accumulation of MVA is lower on lipid substrates (Fig. 2B). Interestingly, when we compared the accumulation of by-products on different carbon sources, we found that the accumulation of citric acid and mannitol on lipid substrates was significantly lower than that on glucose (Fig. 2B). It was consistent with the previous study (Zhao et al. 2015). It is reported that when grown on oleic acid substrate, the metabolism mainly flows to lipogenesis and β-oxidation, while glycolysis, the main source of cytoplasmic NADH, is predominant when grown on glucose (Zhao et al. 2015). This metabolism change may reduce the synthesis of NADH, which may contribute to maintain redox balance and reduce the accumulation of mannitol (Diano et al. 2006; Workman et al. 2013). In addition, high concentration of oleic acid does not cause osmotic stress (Li et al. 2020), which is also a possible reason for reduced mannitol accumulation (Wang et al. 2020). Due to the higher biomass accumulation on lipid substrates, the titer of squalene was also higher on lipid than on glucose. The low accumulation of these by-products indicates that oleic acid is a better substrate for α-farnesene production. Therefore, we used oleic acid as the substrate of F5 strain to produce α-farnesene and optimized it to further improve its α-farnesene production.

### Solely increasing the copy number of FSERG20 did not enhance α-farnesene production obviously

**Fig. 3:**
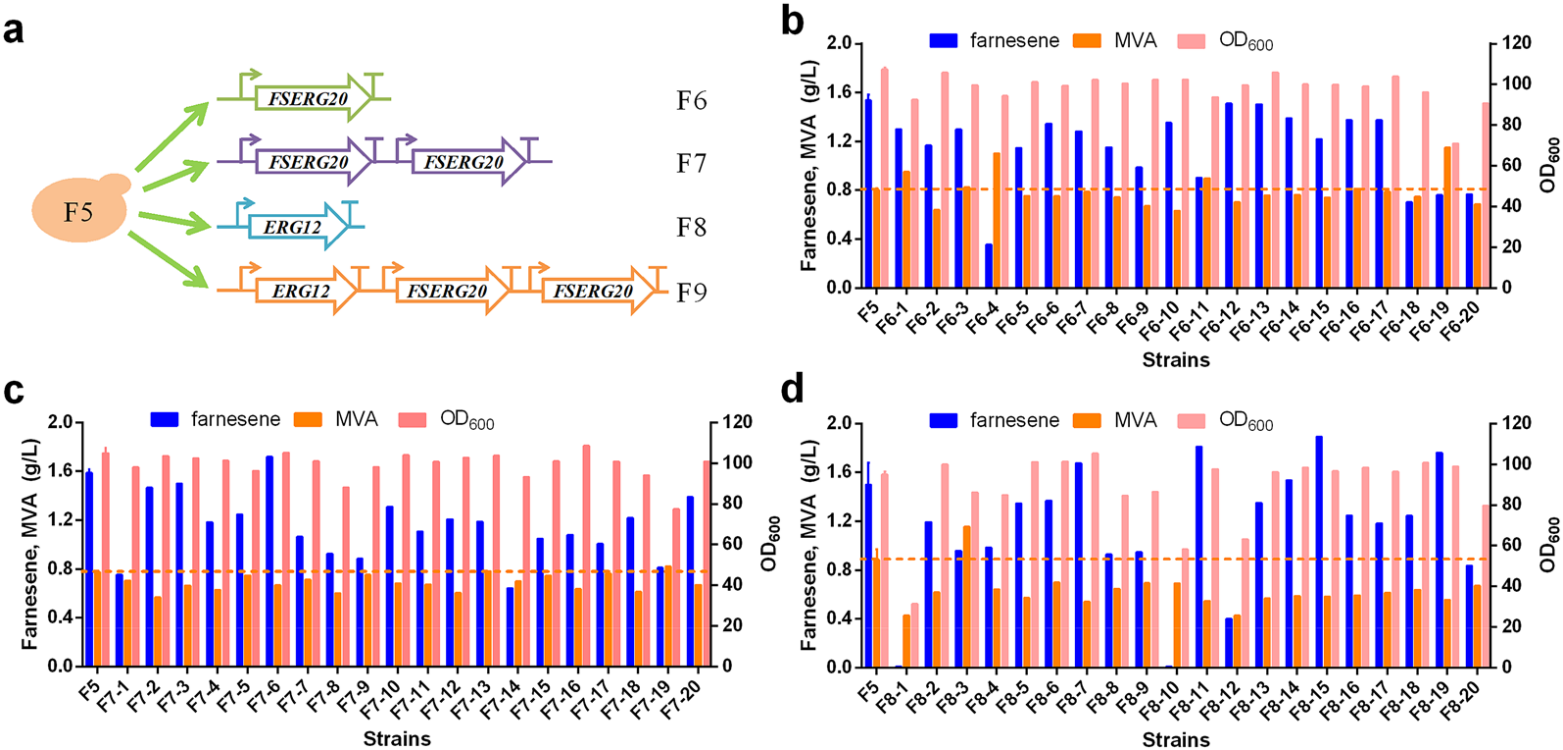
α-Farnesene, mevalonate accumulation and OD_600_ of recombinant strains randomly selected from F6, F7 and F8 libraries. **a** The genetic information of the F6, F7 and F8 strains. **b**–**d** α-Farnesene, mevalonate production and OD_600_ of different transformants randomly selected from F6, F7 and F8 libraries were compared at 96 h, respectively

In our previous study, the strain accumulated 4.5 g/L intermediate metabolite MVA in the late period of fermentation, indicating that there is abundant MVA available for the synthesis of α-farnesene, but it did not flow to the end product due to the restriction of the downstream MVA pathway. Therefore, we attempted to further enhance the strength of the downstream MVA pathway to increase the production of α-farnesene. We increased the copy number of *FSERG20* fusion genes to pull the flux from MVA to α-farnesene. The plasmid containing an expression cassette of *FSERG20* under the control of strong promoter *hp4d* was linearized and transformed into F5 (Fig. 3A). It was randomly integrated into the genome of F5. Because different genomic locations cause the variation of gene expression level (Cui et al. 2019), we randomly selected 20 transformants from the library and detected their titer of α-farnesene, MVA accumulation and the biomass production. Unfortunately, α-farnesene titer was not improved (Fig. 3B). We also constructed the strains containing two *FSERG20* expression cassettes. Only one transformant (F7-6, increased by 7%) from the library showed higher α-farnesene production (Fig. 3C). In addition, the MVA accumulation of most selected transformants was not significantly lower than that of the control F5. We speculated that the pathway from MVA to α-farnesene may not be strong enough; therefore increasing the copy number of *FSERG20* does not enhance α-farnesene production obviously in the F5 strain.

### Overexpression of ERG12 and FSERG20 increased α-farnesene production

Erg12 is the enzyme that catalyzes MVA into phosphomevalonate. Previous studies suggest that Erg12 is also a potential rate limiting step in the MVA pathway (Cao et al. 2017; Marsafari and Xu 2020). Therefore, we detected if the overexpression of *ERG12* could enhance MVA flow to downstream metabolic pathways. We integrated the fragment containing *ERG12* expression cassette into the genome of F5 strain using NHEJ-mediated integration, named F8, and selected 20 transformants for product detection. The overall MVA titer of randomly selected transformants was significantly lower than that of F5 (Fig. 3D). The average titer was about 0.60 g/L, while it was 0.94 g/L in F5. There were four transformants whose α-farnesene titer was higher than that of the F5 strain. The best strain increased α-farnesene titer by 26.8% (F8–15). These results indicate that increasing the expression of *ERG12* does play an important role in promoting MVA to the downstream pathway and Erg12 is the key limiting step to further increase α-farnesene production in the F5 strain. This is consistent with previous reports by Marsafari et al. that showed amorphadiene production was significantly improved through increasing the copy number of *ERG12* in *Y. lipolytica* (Marsafari and Xu 2020), and overexpression of *ERG12* could also increase linalool production in *Y. lipolytica* (Cao et al. 2017). We then inserted two copies of *FSERG20* and one copy of *ERG12* into the genome of F5 to construct F9. Through screening different transformants (Additional file 1: Figure S1), a strain (F9–11) with the α-farnesene titer 2.16 g/L was isolated and the production was 35.8% higher than that of F5 strain. Among 20 transformants, the α-farnesene titer of 6 transformants was higher than that of F5 strain, which reflected that increasing the copy number of *FSERG20* and overexpression of *ERG12* were conducive to increase α-farnesene production.

### Overexpression of VHb to optimizing oxygen utilization enhanced α-farnesene production

**Fig. 4:**
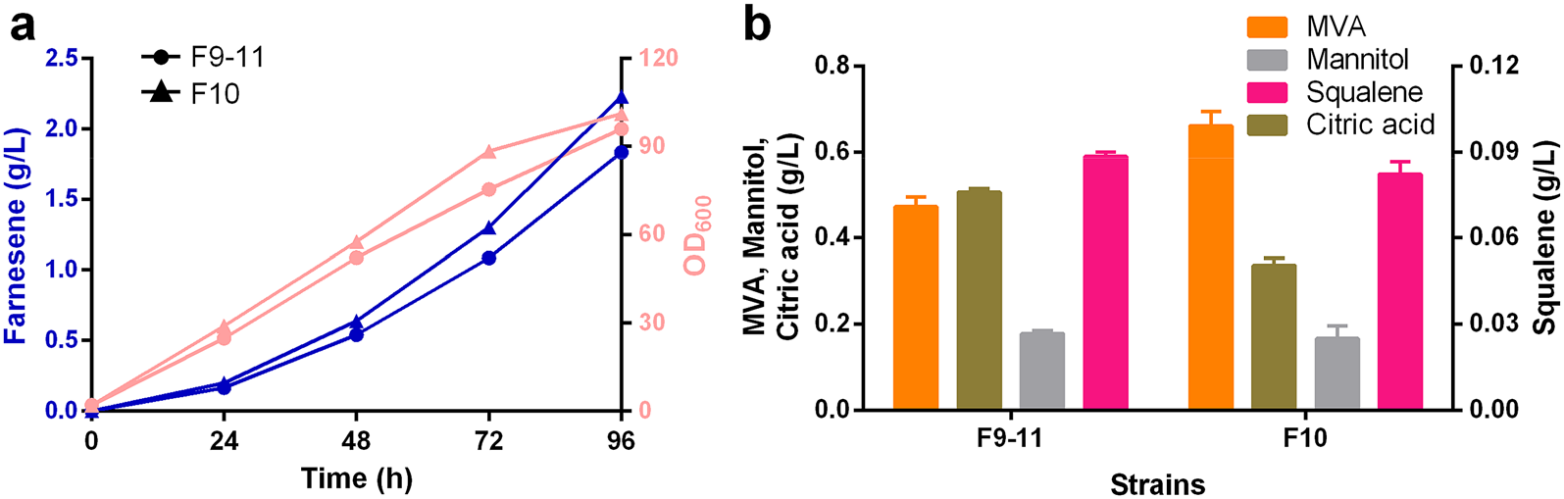
α-Farnesene and other metabolites production in F9-11and F10 using oleic acid as substrate. **a** α-Farnesene accumulation curve of strains F9–11 and F10 during fermentation. **b** Citric acid, mannitol, squalene and mevalonate accumulation at 96 h. Data represent the mean  ±  SD of biological triplicate

Synthesizing one molecule of α-farnesene through the MVA pathway requires nine molecules of ATP, which indicates that α-farnesene synthesis is a high energy-consuming metabolic process. When oleic acid is used as substrate, NADH and FADH_2_ produced by β-oxidation can provide abundant ATP, but it needs oxidative phosphorylation to convert to ATP, which requires oxygen supply. In addition, *Y. lipolytica* is strictly an aerobic yeast, and the oxygen content in the culture environment affects its metabolic process (Akpinar et al. 2011). *Vitreoscilla* hemoglobin (VHb) is a membrane protein. It enhances respiration and energy metabolism by transporting oxygen to the terminal oxidase in cells. In previous studies, heterologous VHb was introduced into *Y. lipolytica* and improved erythritol (Mirończuk et al. 2019) and lipid (Zhang et al. 2019) production, and extracellular enzyme levels (Bhave and Chattoo 2003). Therefore, we introduced a codon-optimized *VHb* expression cassette with the control of *UT8* promoter into the gene locus of *CAN1* (encoding arginine permease) of F9-11 strain by homologous recombination, and obtained F10 strain. The α-farnesene titer of strain F10 reached 2.3 g/L at 96 h in shake flask fermentation, which was 12.7% higher than that of strain F9–11 (Fig. 4A). F10 also accumulated more biomass than F9–11, perhaps because VHb contributes to enhance respiration and makes cells grow more vigorously (Zhang et al. 2019). In addition, VHb expression also decreased citric acid accumulation in F10 than F9–11 (Fig. 4B). F10 accumulated slightly higher MVA, lower mannitol and squalene than F9–11.

### Optimization of fermentation conditions

In metabolic engineering, fermentation parameters such as medium composition, pH, dissolved oxygen and stirring speed have a strong influence on the biosynthesis of the metabolites. Therefore, we optimized the fermentation conditions to maximize the production of α-farnesene synthesized from oleic acid.

We first compared the effects of different initial concentrations of oleic acid on α-farnesene production by adding 20, 35, 50, 65, 80, 95 g/L oleic acid to 50 mL YP medium with 1‰ Tween 80. The results showed that when 20 or 35 g/L of oleic acid was added, the α-farnesene titer and biomass were relatively low and increased with the increase of oleic acid (Additional file 1: Figure S2A). The highest α-farnesene production was achieved when 50 g/L oleic acid was added, and the titer decreased continuously when 65–95 g/L oleic acid was added. The accumulation of MVA increased with the increase of oleic acid, while the biomass kept the highest level when 50 g/L of oleic acid was added. Therefore, the suitable initial addition of oleic acid for strain F10 producing α-farnesene was 50 g/L.

When oleic acid is used as substrate, because of its hydrophobicity, the utilization rate of substrate is low and the mass transfer of air and nutrients in the medium is limited (Liu et al. 2019a). Therefore, non-ionic detergent is usually added to the medium to emulsify the oil matrix and change the properties of the medium. For example, oil degradation was improved by 10–15% after adding oleophilic fertilizer (Zinjarde and Pant 2002). However, the use of these surfactants results in cytotoxicity (Lechuga et al. 2016). Therefore, we optimized the dosage of Tween 80 in the medium. We set six gradients for concentration of Tween 80 as 0.5, 1.0, 1.5, 2.0, 2.5, and 3.0‰ in the final medium. There was no significant difference in α-farnesene production, MVA and biomass accumulation at 96 h of fermentation within the selected gradient range (Additional file 1: Figure S2B). It is possible that the surfactant secretion of *Y. lipolytica*, such as liposan, eliminated the effect of different Tween 80 dosage when growing on oil (Gonçalves et al. 2014). We chose the 1.0‰ working concentration of Tween 80.

Metal ions are involved in many biological processes, including signal transduction, regulation of enzyme activity, maintenance of cell stability, protection of cells, etc., which is an important factor affecting microbial growth and metabolite biosynthesis (Jernejc and Legisa 2002; Misra et al. 2019). In particular, farnesene synthetases, similar to other terpenoid synthetases, contain Mg^2+^ binding site in their domains, and their activities are also affected by the type and concentration of metal ions (Picaud et al. 2005). Therefore, we added 5 mM different divalent cations to the medium, including Ca^2+^, Cu^2+^, Fe^2+^, Mg^2+^, Mn^2+^ or Zn^2+^, to explore the effect on α-farnesene synthesis. The results showed that α-farnesene production of the experimental group adding Mg^2+^ was basically similar to that of the control group without adding ions (Additional file 1: Figure S2C). α-Farnesene titer was reduced at different degrees after adding other ions. Maybe the original content of metal ions in the medium was enough to meet the requirement to maintain the growth and metabolic activities of cells. Interestingly, the accumulation of MVA increased by 41.5% and 135.6%, respectively, when adding Fe^2+^ and Zn^2+^. Although this accumulation is unfavorable to the F10 strain, these two ions can be added to other strains that synthesize products by the MVA pathway to increase the supply of intermediate products.

**Fig. 5:**
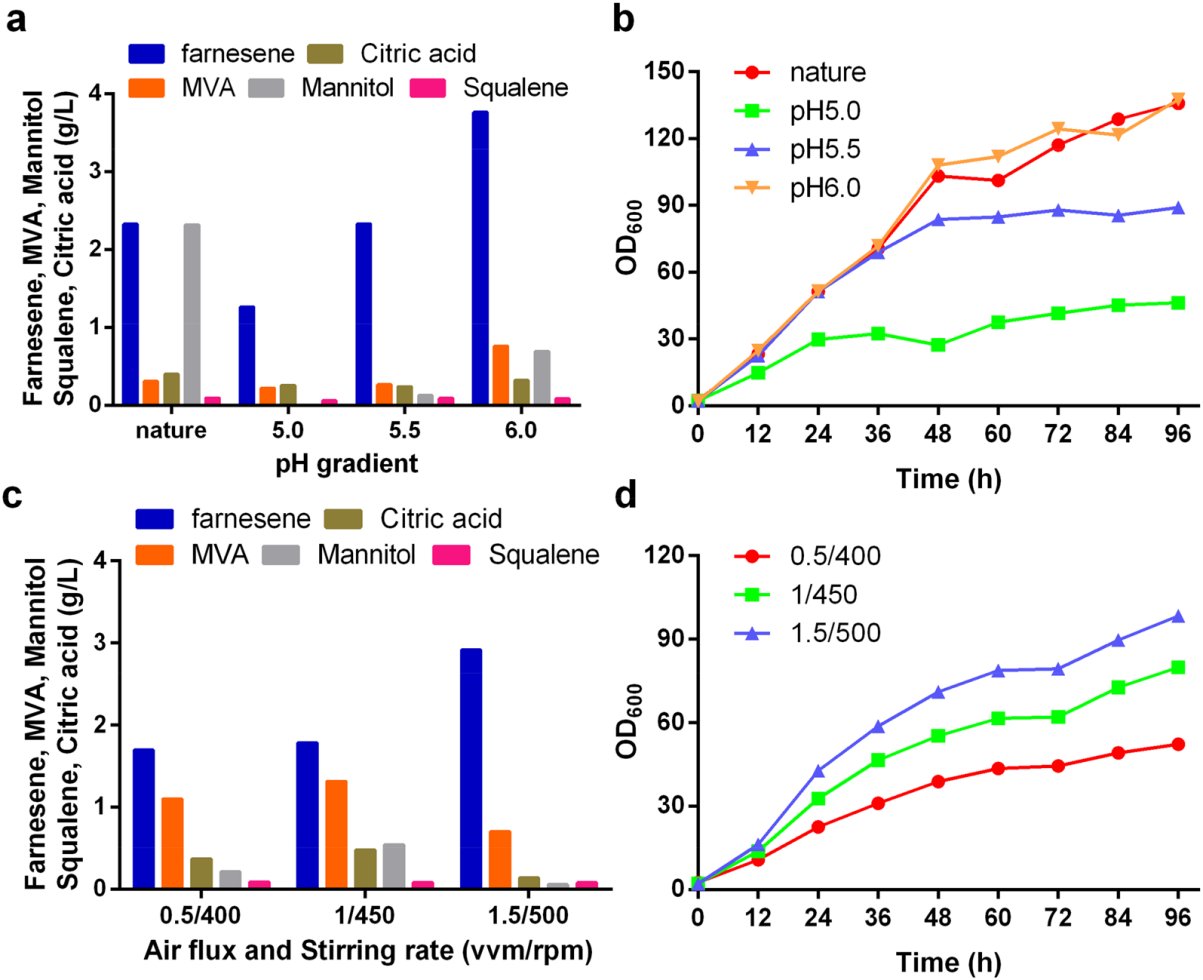
Optimization of pH, air flux and stirring rate for fermentation of strain F10 in a bioreactor. **a** Titer of α-farnesene and metabolite accumulation at different pH. **b** Growth curve at different pH during fermentation. **c** Titer of α-farnesene and metabolite accumulation at different air flux and stirring rate. **d** Growth curve at different air flux and stirring rate during fermentation. α-Farnesene and metabolites were detected after 96 h of fermentation in a 1-L bioreactor containing 800 mL YP medium with oleic acid. This experiment was performed with *n*  =  1

Then, we explored the effects of pH, dissolved oxygen and stirring speed on α-farnesene production in a 1 L fermenter. The pH of the fermentation broth was controlled at 5.0, 5.5, 6.0 or in a natural state without pH regulation. F10 had the highest α-farnesene titer of 3.76 g/L and OD_600_ of 135 at pH 6.0 with MVA accumulation at 0.76 g/L (Fig. 5A, B). However without adjusting pH, the initial pH of the medium was 5.8, close to 6.0, and changed to 6.2 after 96 h, and the biomass also reached to 135, but the α-farnesene titer was only 2.32 g/L. In the meanwhile, the mannitol accumulated to 2.3 g/L, which was 2.3-fold higher than the titer at pH 6.0. It is suggested that the metabolism of MVA pathway may be more vigorous or the activity of α-farnesene synthetase may be higher under constant optimal pH. In addition, the by-product accumulation of citric acid and squalene in all experimental groups was at a low level. We finally chose pH 6.0 as the optimal pH for α-farnesene synthesis of strain F10.

On the basis of pH 6.0, we optimized the air flux and stirring rate in 1 L fermentor under three combination conditions of 0.5/400, 1.0/450 and 1.5/500 (vvm/rpm). With the increase of air flux and stirring rate, more biomass was accumulated during fermentation (Fig. 5D). The highest α-farnesene titer was 2.9 g/L at 1.5 vvm air flux and 500 rpm stirring rate with lower MVA, citric acid, mannitol and squalene accumulation than that of other experimental groups (Fig. 5C). Because *Y. lipolytica* is strictly an aerobic yeast, a high dissolved oxygen medium is conducive to metabolic activities and product synthesis. Finally, we chose 1.5/500 (vvm/rpm) as the best condition for α-farnesene synthesis.

**Fig. 6:**
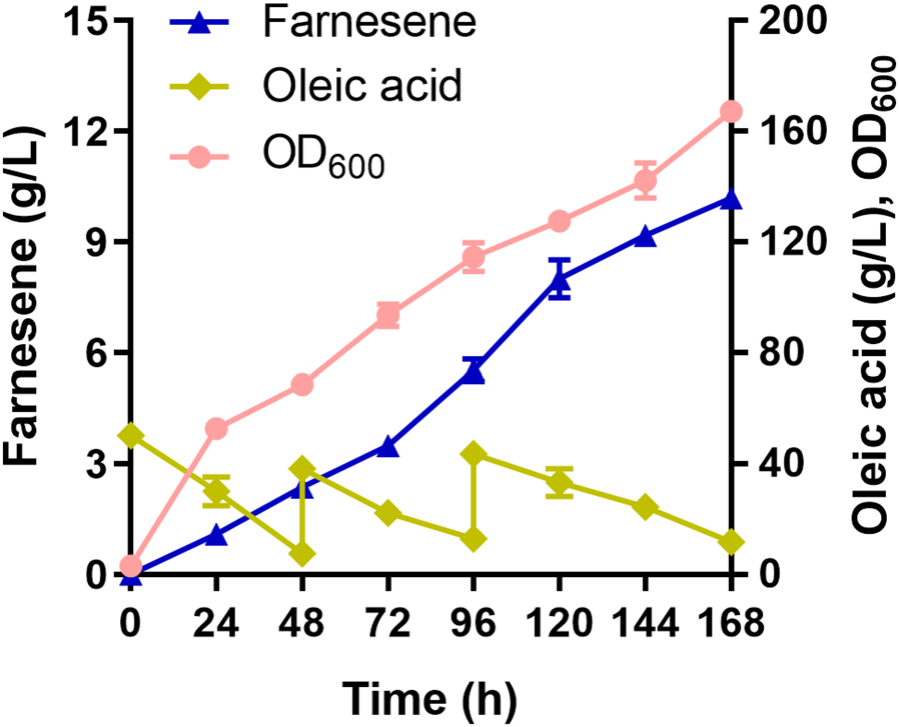
α-Farnesene production, oleic acid consumption and growth of strain F10 in fed-batch cultivations. The cultivation was carried out in a 5-L bioreactor containing 4 L YP medium with oleic acid at pH 6.0, 1.5 vvm air flux, and 500 rpm stirring rate. Oleic acid was fed when it was lower than 10 g/L. The error bars represent standard deviations of duplicate cultivations

Under the optimal conditions for α-farnesene synthesis by strain F10, including 50 g/L initial oleic acid concentration, 1‰ Tween 80 dosage, no additional metal ion addition, pH 6.0, 1.5 vvm air flux and 500 rpm stirring rate, we carried out fed batch fermentation in 5 L bioreactor. During the fermentation process, the production of α-farnesene reached 10.2 g/L at 168 h and showed a trend of increasing (Fig. 6), which was higher than that of the F5 strain of 7.4 g/L at 168 h (Liu et al. 2019b). It is worth noting that the α-farnesene conversion rate of strain F10 is 0.1 g/g oleic acid, while that of strain F5 is only 0.038 g/g glucose, which shows that strain F10 has the great advantage of using oleic acid to produce α-farnesene. In the final fermentation broth, OD_600_ reached 167.3, MVA accumulation was 2.2 g/L, and by-products citric acid, mannitol and squalene accumulation were at a low level, reached 0.84 g/L, 0.31 g/L and 0.11 g/L respectively (Additional file 1: Figure S3), indicating that F10 had good performance of synthesizing α-farnesene from oleic acid.

### Exploration of α-farnesene synthesis capability on waste cooking oils

Waste cooking oil (WCO) is an inevitable product in the catering industry. It is reported that the global annual output of WCO exceeds 29 million tons (Maddikeri et al. 2012). Improperly handling of WCO will cause environmental and ecological problems (Lopes et al. 2019). Traditionally, WCO has been used in soap making, as an additive in livestock ingredients or as a cheap detergent (Domínguez et al. 2010). However, it also has low economic benefits, and the harmful substances in WCO may also be transferred to the human body through the food chain (Lam et al. 2016). New applications have also been developed in recent years. For example, WCO was used as the substrate of biodiesel production. As a nutrient of fermentation medium, WCO provides carbon source for microorganisms to produce various products. For example, using WCO as substrate, the engineered *Y. lipolytica* strain produced limonene, bisabolene, citric acid and erythritol, which increased the economic benefits and realized green development (Liu et al. 2015, 2019a; Pang et al. 2019; Zhao et al. 2021). These researches demonstrated that *Y. lipolytica* has potential in the conversion of WCO to valuable products with economical and sustainable advantage.

We explored the α-farnesene synthesis ability of F10 strain on WCO carbon source. The WCO we used was from school canteen and was the frying waste of soybean oil. To investigate the optimized concentration of WCO (Aggelis et al. 2003), we added 35, 50, 65, 80 or 95 g/L WCO to 50 mL YP medium with 1‰ Tween 80. At 96 h of fermentation, MVA accumulation increased with the increase of WCO content, but α-farnesene production had no significant difference, about 1.6 g/L (Additional file 1: Figure S4A). It seems WCO was in excess for α-farnesene production. We also found that the titer of α-farnesene was lower when using WCO as carbon source. The impurities in WCO may be the reasons for the lower α-farnesene titer on WCO than that on oleic acid. Using WCO, OD_600_ can reach 93, slightly lower than that with oleic acid. Our results showed that the F10 strain has good tolerance to the harmful components of WCO and makes it a suitable host for use of waste oil to produce high value-added products (Moftah et al. 2013; Zinjarde et al. 2014). We also explored the α-farnesene synthesis ability of F10 strain on other major edible oils at 50 g/L final concentration. In shake flasks, the α-farnesene productions were 1.74, 1.52, 1.78 and 1.76 g/L on olive oil, soybean oil, palm oil and rapeseed oil, respectively, at 96 h (Additional file 1: Figure S4B). The results indicated that F10 was not only suitable for WCO from soybean oil, but also suitable for WCO from other kinds of oil or mixed oil.

## Conclusions

In this study, we optimized the α-farnesene producing strain and found that Erg12 was the key limiting factor to further increase the production of the strain. The α-farnesene production of the recombinant strain was further improved by increasing the copy number of *FSERG20*. To make the recombinant strain more suitable for α-farnesene synthesis on cheap oleic acid substrate, we expressed heterologous VHb to improve its oxygen utilization and energy metabolism. Combined with the optimization of fermentation conditions, the α-farnesene synthesis ability of the recombinant strain was optimized. Finally, the α-farnesene titer and yield reached 10.2 g/L and 0.1 g/g oleic acid, respectively, in 5 L fermentor at 168 h. The accumulation of by-products or MVA was significantly lower than that on glucose. In addition, we also explored the α-farnesene synthesis ability on WCO and other edible oils, to realize the green production from waste utilization. Our results showed the potential of *Y. lipolytica* to synthesize high value-added products using cheap WCO or other waste oil substrate.

## Materials and methods

### Strains, media, and culture conditions

For plasmid construction and proliferation, *Escherichia coli* strain DH5α was employed and cultured in Luria–Bertani (LB) complete medium (5 g/L yeast extract, 10 g/L tryptone and 10 g/L NaCl) at 37 °C, 200 rpm. Agar (20 g/L) was used as solid medium, and ampicillin (50 mg/L) was added to LB when necessary. *Y. lipolytica* strains were cultured at 30 °C, 220 rpm in YPD medium (20 g/L glucose, 20 g/L tryptone, 10 g/L yeast extract). Minimal SD Base (TaKaRa, Beijing, China) with arginine dropout amino acid mixes was used for transformant selection. Canavanine (60 mg/L; Sigma, Shanghai, China) and nourseothricin (0.5 g/L; Sigma, Shanghai, China) were added to YPD or SD medium when necessary. *Y. lipolytica* strains used in this study are listed in Table 1.

**Table 1:** Strains used in this study

Name	Description	Reference
F5	AHH12^a^ harboring linearized plasmid JMP-hyg-FSERG20-IDI-ERG12, 114-GPPS-ERG8-ERG19, and YLEP-URA-FSERG20	(Liu et al. 2019b)
F6	F5 harboring linearized plasmid pki-nat-FSERG20	This study
F7	F5 harboring linearized plasmid pki-nat-2FSERG20	This study
F8	F5 harboring linearized plasmid YLEP-nat-ERG12	This study
F9	F5 harboring linearized plasmid pki-nat-2FSERG20-ERG12	This study
F10	F9, *Can1*::*UT8-VHb-CYC1*	This study

^a^AHH12 is derived from *Y. lipolytica* PO1f

### Plasmids and strains construction

The primers used for plasmids construction were synthesized by TsingKe (Beijing, China). DNA fragments were obtained by polymerase chain reaction (PCR) using DNA polymerase (Vazyme, Nanjing, China). The restriction enzymes used for plasmids construction and linearization were purchased from ThermoFisher Scientific (Shanghai, China). Gibson assembly was used for plasmid construction (Gibson et al. 2009). All the primers and plasmids used in this study are shown in Additional file 1: Table S1 and Table 2, respectively.

**Table 2:** Plasmids used in this study

Name	Description	Reference
pki-nat	*Y. lipolytica* integrative vector, *hp4d* promoter with *XPR2* terminator, *TEF* promoter with *CYC1* terminator, nourseothricin selection marker	This study
YLEP-nat	*Y. lipolytica* episomal vector, *UT8* promoter with *CYC1* terminator, nourseothricin selection marker	This study
pki-nat-FSERG20	pki-nat vector containing a fusion gene of codon-optimized *FS* and *ERG20* with GGGS linker between *FS* and *ERG20* (*FSERG20*) under *hp4d* promoter	This study
pki-nat-2FSERG20	pki-nat-FSERG20 vector containing another *FSERG20* under *TEF* promoter	This study
pki-nat-2FSERG20-ERG12	pki-nat-2FSERG20 vector containing *UT8-ERG12-CYC1* under *TEF* promoter	This study
YLEP-nat-ERG12	YLEP-nat vector containing *ERG12*	This study
YLEP-VHb	YLEP-nat vector containing *VHb*	This study

For *Y. lipolytica* transformants selection, the selection marker *URA3* and *LEU2* in plasmids pki-2 and YLEP-Leu were replaced by nourseothricin acetyltransferase gene to construct pki-nat and YLEP-nat, respectively. *FSERG20* was α-farnesene synthase gene (*FS*) from apple seeds and codon optimized according to the codon preference of *Y. lipolytica* (GenBank: MZ343331), fused with FPP synthase gene (*ERG20*, GenBank: YALI0_E05753g) via a GGGS amino acid linker. To construct one or two expression cassettes of *FSERG20*, we inserted *FSERG20* under the control *hp4d* and *TEF* promoter in pki-nat plasmid to form pki-nat-FSERG20 and pki-nat-2FSERG20. Plasmids pki-nat-FSERG20 and pki-nat-2FSERG20 were linearized by SspI, transformed and integrated into the genome of F5 strain via non-homologous end-joining (NHEJ)-mediated integration. Transformants were selected on YPD plates with nourseothricin and were verified by clone PCR to generate strains named F6 and F7, respectively. Twenty transformants of each transformant plates were selected randomly and fermented in shake flasks to detect α-farnesene production. To overexpress mevalonate kinase gene (*ERG12*, GenBank: YALI0_B16038g), *ERG12* gene with *UT8* promoter and *CYC1* terminator were inserted into plasmid YLEP-nat to construct YLEP-nat-ERG12. F8 strain library was obtained by transforming linearized YLEP-nat-ERG12 into F5 strain using the same method. To obtain strain F9, we constructed a plasmid containing *ERG12* and two *FSERG20* expression cassettes with nourseothricin acetyltransferase gene as selection marker and named it as pki-nat-2FSERG20-ERG12. The plasmid was linearized by SspI and integrated into the genome of F5 strain via NHEJ-mediated integration. Transformants were fermented in shake flasks. Hemoglobin coding base from *Vitreoscilla stercoraria* was optimized according to the codon preference of *Y. lipolytica* (*VHb*, GenBank: MZ343332). *VHb* was placed under the control of the *UT8* promoter of YLEP-nat to form the YLEP-VHb plasmid. The *UT8-VHb-CYC1* fragment was combined with upstream and downstream homologous arm of canavanine amino acid permease coding gene (*can1*, GenBank: YALI0_B19338g) by overlap PCR. The recombined fragment was transformed F9 strain and replaced *can1* gene by homologous recombination. The right transformants were screened by SD-Arg plates with canavanine and checked by clone PCR, forming *VHb* overexpression strain named F10. The DNA fragments were transformed into *Y. lipolytica* using the lithium acetate method (Chen et al. 1997). *FS* and *VHb* were synthesized by GENERAL BIOSYSTEMS (Anhui, China).

### Fermentation and optimization of fermentation conditions

For shake flask cultivations, the single colonies were inoculated in 24-well plate containing 1.5 mL YPD medium. Then 1–1.5 mL of the culture was inoculated in a 300-mL flask containing 50 mL medium and cultured for 96 h at 30 ℃ and 220 rpm. Dodecane (10%) was added to collect α-farnesene. YP (20 g/L tryptone, 10 g/L yeast extract) was used as basic components of fermentation medium, and different carbon sources including glucose, oleic acid, palm oil, soybean oil, rapeseed oil, olive oil and waste cooking oils were added to YP when necessary. Tween 80 was added to the medium as an emulsifier when lipid carbon sources were used. Different fermentation conditions were optimized at shake flask level, including the initial amount of oleic acid, the concentration of emulsifier and the addition of different kinds of divalent cations. The initial dosage of oleic acid was set as 20, 35, 50, 65, 80, and 95 g/L. The final concentration of emulsifier was set as 0.5, 1.0, 1.5, 2.0, 2.5 or 3.0‰. Cations were added to the medium in the form of sulfates and the species of cations include Ca^2+^, Cu^2+^, Fe^2+^, Mg^2+^, Mn^2+^ and Zn^2+^ with 5 mM final concentration.

At the bioreactor level, to activate the preserved α-farnesene synthesis ability of strain F10, it was inoculated in a test tube containing 5 mL YPD medium and then 1 mL culture was inoculated in a 300-mL flask containing 50 mL YPD medium. The second pre-culture (5 mL) was transferred in a 500-mL flask containing 100 mL YPD medium cultured for the final fermentation seeds. All the above culture conditions were 30 ℃ and 220 rpm for 24 h. The prepared culture was transferred in 1 L or 5 L bioreactor with 10% inoculum and 10% dodecane. The fermentation medium is YP with 50 g/L oleic acid. The working volumes of 1 L and 5 L bioreactor were 700 mL and 4 L, respectively. Different cultivation conditions were compared, including variation of pH and air flux combining with stirring rate in 1 L bioreactor. The pH was set at 5.0, 5.5, 6.0 or not adjusted. The combinations of air flux and stirring rate were set at 0.5/400, 1.0/450, or 1.5/550 (vvm/rpm). When the remaining oleic acid content in the medium was not enough to maintain for 12 h, 50% oleic acid was added during fermentation.

### ***Analysis of OD***_***600***_***, mevalonate, citric acid and mannitol***

Biomass in the fermentation broth was detected by a UV-1800 spectrophotometer (Shimadzu, Japan) at 600 nm after appropriate multiple diluting. To analyze the accumulation of mevalonate, citric acid and mannitol during fermentation, the fermentation broth (1 mL) was taken and centrifuged and the supernatant was filtered using a 0.22-μm filter subsequently. When using lipid substrates, the supernatant needs to be treated twice with 200 μL *n*-hexane before filtration to remove the remaining lipid. High-performance liquid chromatography system (HPLC) equipped with an Aminex HPX-87H column (BioRad, Inc., Hercules, CA) and a refractive index detector, H_2_SO_4_ (5 mM) mobile phase with flow rate 0.6 mL/min at 65 °C was used for qualitative detection of mevalonate, citric acid and mannitol.

### Analysis of oleic acid in the fermentation broth

To detect the remaining oleic acid content in the fermentation broth, 1 mL of fermentation broth was taken and centrifuged to remove the cells. The oleic acid in the supernatant was extracted with *n*-hexane. Oleic acid was separated through evaporating *n*-hexane at 65 °C. The extracted oleic acid was methylated with 1% methanol sulfate (v:v) to obtain methyl oleate, and its content was determined by gas chromatography (GC; Agilent Technologies, Santa Clara, CA) with an Rtx-5 capillary column (30.0 m, 0.25 mm ID, 0.25 μm df; RESTEK, USA) and a flame ionization detector (FID). The detection conditions are as follows: the temperatures of the injector and the detector were set at 280 °C and 290 °C, respectively. The oven temperature was kept at 50 °C for 2 min and then ramped to 140 °C at 30 °C/min and continued to heat up to 280 °C at 10 °C/min and held for 5 min.

### Analysis of α-farnesene

To analyze the concentration of α-farnesene during fermentation, the dodecane was collected from 2 mL fermentation broth by centrifuging for 2 min at 13,000 rpm. Then the dodecane was injected into GC with an Rtx-5 capillary column (30.0 m, 0.25 mm ID, 0.25 μm df; RESTEK, USA) and a flame ionization detector (FID). The detection conditions are as follows: the temperatures of the injector and the detector were set at 280 °C and 290 °C, respectively. The oven temperature was kept at 80 °C for 1 min, then ramped to 250 °C at 10 °C/min and held for 1 min, and then to 280 °C at 10 °C/min and held for 2 min.

### Supplementary Information

**Additional file 1: Figure S1.** α-Farnesene, mevalonate accumulation and OD_600_ of recombinant strains randomly selected from F9 library. Data were detected after 96 h of fermentation in 300-mL shaken flasks containing 50 mL YP medium with oleic acid. Date of transformants were performed with *n* = 1, while date of F5 strain represent the mean±SD of biological triplicates. **Figure S2. **Optimization of medium for fermentation of F10 strain in shake flasks. a Initial concentration of oleic acid. b Concentration of Tween 80 (‰). c Different cations. All data represents the mean ± SD of biological triplicates**. Figure S3.** The mevalonate and by-products production in 5 L fermentor under the optimum conditions of F10 strain. The error bars represent standard deviations of duplicate cultivations. **Figure S4.** α-Farnesene production on WCO and different oil substrates. a α-Farnesene, mevalonate accumulation and OD_600_ of F10 strain under different WCO initial addition at 96 h. b α-Farnesene accumulation and OD_600_ of F10 strain on different oil types at 96 h. Data represents the mean ± SD of biological triplicates. **Table S1.** Primers used in this study.

## Data Availability

All data generated or analyzed during this study were included in this published article [and its Additional files].

## Abbreviations

NHEJ: Non-homologous end-joining
FFA: Free fatty acid
FA-CoA: Fatty acid-CoA
MVA: Mevalonate
IPP: Isopentenyl diphosphate
FPP: Farnesyl diphosphate
ERG12: Mevalonate kinase
ERG20: FPP synthase
FS: Codon-optimized α-farnesene synthase
VHb: Hemoglobin from *Vitreoscilla stercoraria*
