# Supplementary material for: α-Farnesene production from lipid by engineered Yarrowia lipolytica
Source: Bioresour Bioprocess. 2021 Aug 23;8(1):78. doi: 10.1186/s40643-021-00431-0 (PMC10991571; doi:10.1186/s40643-021-00431-0)
Supplement: Supplementary file 1 — Additional file 1: Figure S1. α-Farnesene, mevalonate accumulation and OD600 of recombinant strains randomly selected from F9 library. Data were detected after 96 h of fermentation in 300-mL shaken flasks containing 50 mL YP medium with oleic acid. Date of transformants were performed with n = 1, while date of F5 strain represent the mean±SD of biological triplicates. Figure S2. Optimization of medium for fermentation of F10 strain in shake flasks. a Initial concentration of oleic acid. b Concentration of Tween 80 (‰). c Different cations. All data represents the mean ± SD of biological triplicates. Figure S3. The mevalonate and by-products production in 5 L fermentor under the optimum conditions of F10 strain. The error bars represent standard deviations of duplicate cultivations. Figure S4. α-Farnesene production on WCO and different oil substrates. a α-Farnesene, mevalonate accumulation and OD600 of F10 strain under different WCO initial addition at 96 h. b α-Farnesene accumulation and OD600 of F10 strain on different oil types at 96 h. Data represents the mean ± SD of biological triplicates. Table S1. Primers used in this study. [file 40643_2021_431_MOESM1_ESM.docx]

**Bioresources and Bioprocessing**

**Supplementary informations**

**α-farnesene production from lipid by engineered *Yarrowia lipolytica***

Yinghang Liu^1^, Zhaoxuan Wang^1^, Zhiyong Cui^1^, Qingsheng Qi^1, 2^*, Jin Hou^1^*

^1^ State Key Laboratory of Microbial Technology, Shandong University, Qingdao 266237, P. R. China

^2^CAS Key Lab of Biobased Materials, Qingdao Institute of Bioenergy and Bioprocess Technology, Chinese Academy of Sciences, Qingdao 266101, P. R. China

* Corresponding author: Prof. Jin Hou, email: [houjin@sdu.edu.cn](mailto:houjin@sdu.edu.cn), Prof. Qingsheng Qi, email: [qiqingsheng@sdu.edu.cn](mailto:qiqingsheng@sdu.edu.cn), State Key Laboratory of Microbial Technology, Shandong University, Binhai Road 72, Qingdao 266237, P. R. China


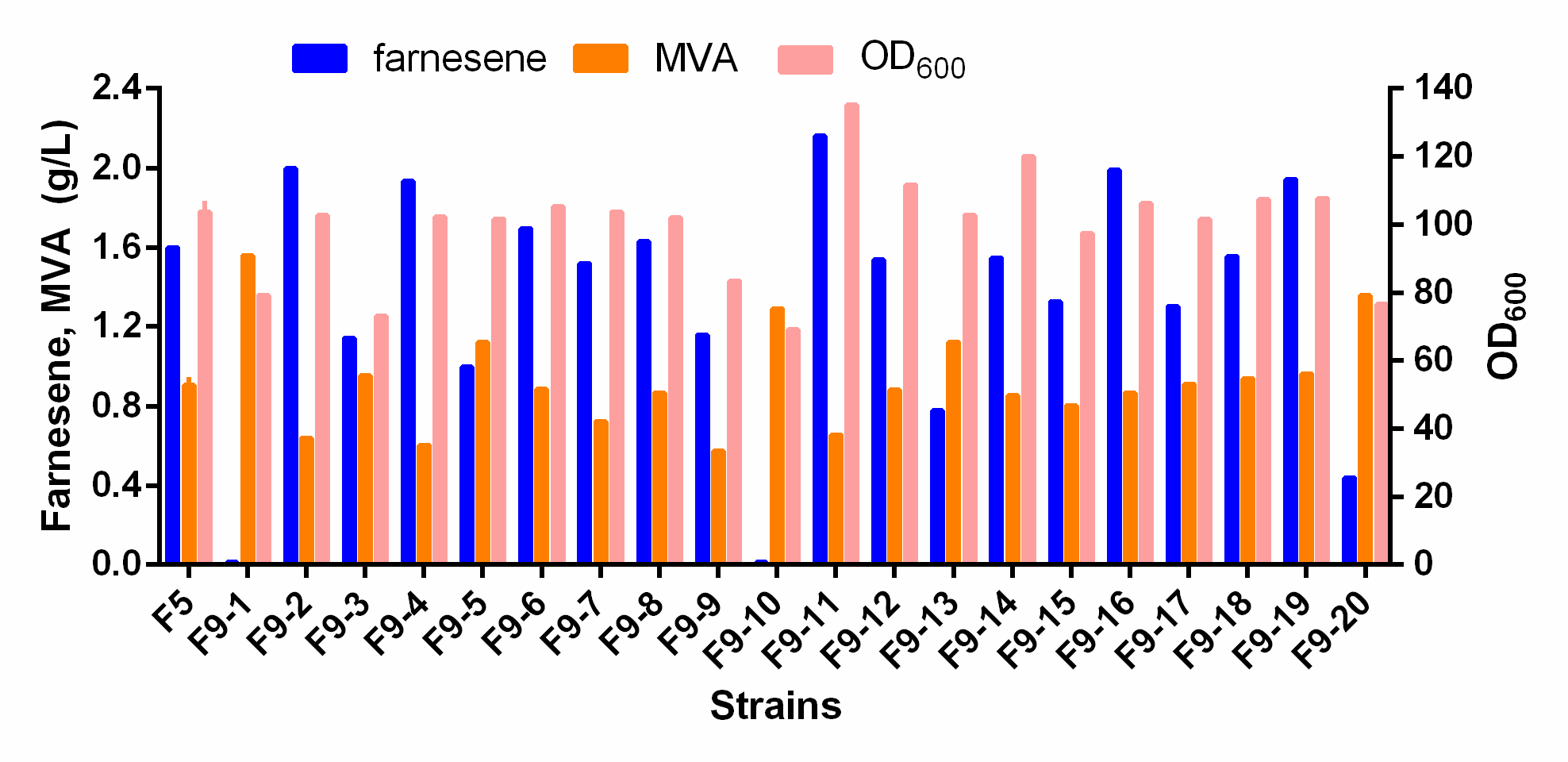


**Fig. S1 α-Farnesene, mevalonate accumulation and OD_600_ of recombinant strains randomly selected from F9 library.** Data were detected after 96 h of fermentation in 300-mL shaken flasks containing 50 mL YP medium with oleic acid. Date of transformants were performed with n=1, while date of F5 strain represent the mean±SD of biological triplicates


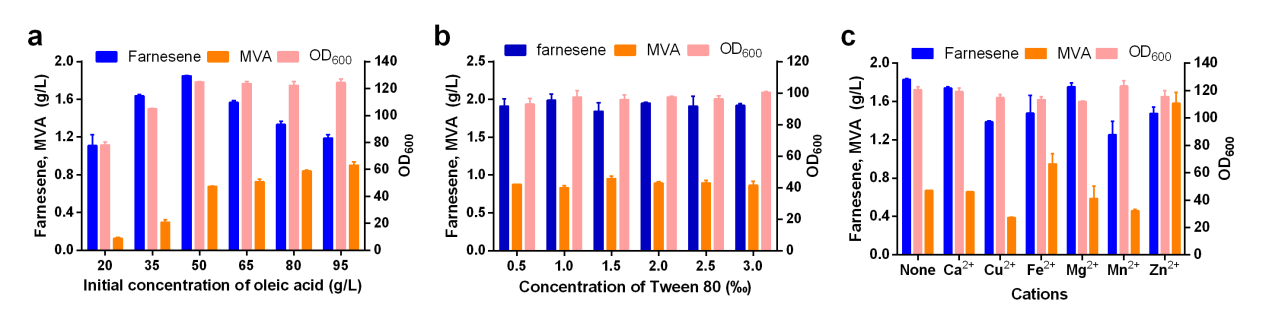


**Fig. S2 Optimization of medium for fermentation of F10 strain in shake flasks.** (a) Initial concentration of oleic acid. (b) Concentration of Tween 80 (‰). (c) Different cations. All data represents the mean±SD of biological triplicates


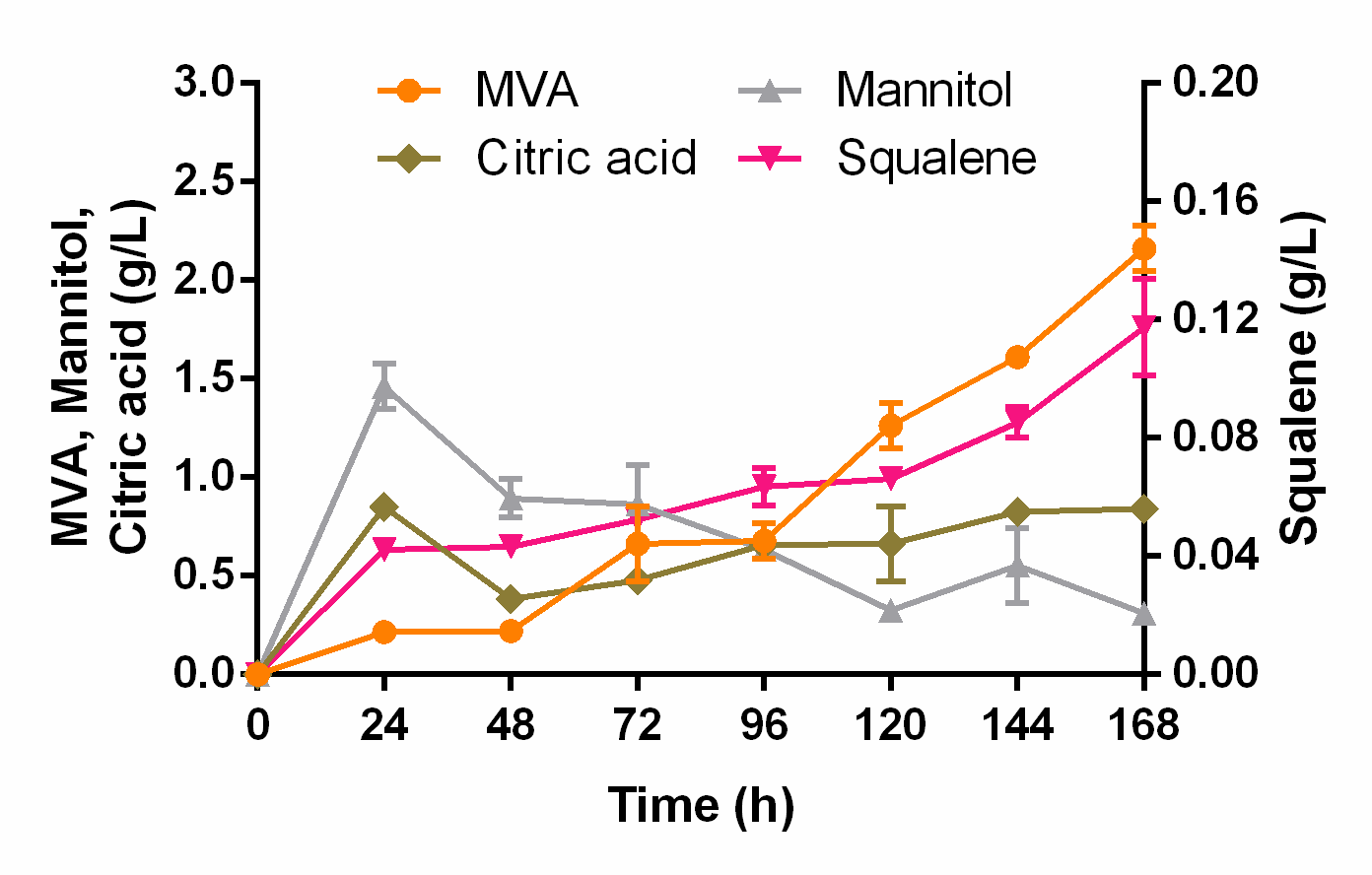


**Fig. S3 The mevalonate and by-products production in 5 L fermentor under the optimum conditions of F10 strain.** The error bars represent standard deviations of duplicate cultivations





**Fig. S4 α-Farnesene production on WCO and different oil substrates.** (a) α-Farnesene, mevalonate accumulation and OD_600_ of F10 strain under different WCO initial addition at 96 h. (b) α-Farnesene accumulation and OD_600_ of F10 strain on different oil types at 96 h. Data represents the mean±SD of biological triplicates

**Supplementary Table S1. Primers used in this study**

| Name | Sequence (5’-3’) | Purpose |
| --- | --- | --- |
| pki-nat-F | cagtctcctcttcaccaccaaaatggacatggcatagacata | Amplifying nourseothricin acetyltransferase gene |
| pki-nat-R | tacatatccatagtctaaccttactactttggatgatactg |  |
| pki-F | aggttagactatggatatgta | Amplifying pki-2 vector |
| pki-R | tttggtggtgaagaggagactg |  |
| YLEP-F | tgtggatgtgtgtggttgtatgtg | Amplifying YLEP-Leu vector |
| YLEP-R | gtcgtttctacgacgcattgatg |  |
| hp4d-FSErg20-F | tccacgtgggaaccgcgatcgcatggaattccgagtgcacct | Amplifying *FSERG20* |
| hp4d-FSErg20-R | aggccatggaggtacgcgatcgcctacttctgtcgcttgtaaatc |  |
| TEF-FSErg20-F | gaggtgcactcggaattccatatttaaatctgcggttagtac | Amplifying *FSERG20* |
| TEF-FSErg20-R | actaattacatgaatttaaatctacttctgtcgcttgtaaat |  |
| Erg12(ut8)-F | taagaatcattcaaaggttcgaaatggactacatcatttcggc | Amplifying *ERG12* |
| Erg12(CYC1)-R | cataactaattacatgattcgaactaatgggtccagggaccga |  |
| utp-F | aacccggtctctaagctagcggtaccaaggaagcatgcggt | Amplifying  *ut8-ERG12-CYC1* fragment |
| utp-R | gcatgcacgcgtatcgataagcaaattaaagccttcgagcg |  |
| Can1-P1N | ctggaatggtaaacacacgag | Amplifying Upstream homologous arm of *can1* |
| Can1-up-R | tgtggtcggatggatggagacggc |  |
| Can1-down-F | gaagatcggggattcccgacgctc | Amplifying Downstream homologous arm of *can1* |
| Can1-P6N | attccttcgccagtcattgtg |  |
| Can1-ut8-F | gccgtctccatccatccgaccacaggtaccaaggaagcatgcggt | Amplifying *ut8-VHb-CYC1* fragment |
| Can1-CYC1-R | gagcgtcgggaatccccgatcttcgcaaattaaagccttcgagcg |  |
